# Correlation between coronary heart disease severity and subsequent chronic rhinosinusitis severity: A retrospective cohort study

**DOI:** 10.7150/ijms.86601

**Published:** 2023-08-06

**Authors:** Ke-Hsin Ting, Yen-Ting Lu, Chung-Han Hsin, Chia-Yi Lee, Jing-Yang Huang, Shun-Fa Yang, Ming-Hong Hsieh

**Affiliations:** 1 Division of Cardiology, Department of Internal Medicine, Changhua Christian Hospital, Yunlin Branch, Yunlin, Taiwan.; 2 Institute of Medicine, Chung Shan Medical University, Taichung, Taiwan.; 3 Department of Post-Baccalaureate Medicine, College of Medicine, National Chung Hsing University, Taichung, Taiwan.; 4 Department of Otolaryngology, Chung Shan Medical University Hospital, Taichung, Taiwan.; 5 School of Medicine, Chung Shan Medical University, Taichung, Taiwan.; 6 Department of Otolaryngology, St. Martin De Porres Hospital, Chiayi, Taiwan.; 7 Department of Ophthalmology, Nobel Eye Institute, Taipei, Taiwan.; 8 Department of Ophthalmology, Jen-Ai Hospital Dali Branch, Taichung, Taiwan.; 9 Department of Medical Research, Chung Shan Medical University Hospital, Taichung, Taiwan.; 10 Department of Psychiatry, Chung Shan Medical University Hospital, Taichung, Taiwan.

**Keywords:** coronary heart disease, chronic rhinosinusitis, severity, male, epidemiology

## Abstract

Coronary heart disease (CHD) is associated with the development of several diseases. This retrospective population-based cohort study investigated the association between CHD severity and subsequent chronic rhinosinusitis (CRS) of varying severity. We used data from Taiwan's National Health Insurance Research Database. CHD was categorized as severe if treated using a coronary artery bypass graft (CABG) and as mild if treated with percutaneous coronary intervention (PCI). The primary outcome of this study was the development of CRS or severe CRS treated using functional endoscopic sinus surgery. Cox proportional hazards regression was used to calculate adjusted hazard ratios (aHRs) and 95% confidence intervals (CIs) for CRS and severe CRS in different patient groups. We included 31,784 patients who received PCI surgery (the CHD-PCI group) and 15,892 patients who received CABG surgery (the CHD-CABG group). A total of 813 and 482 episodes of CRS occurred in the CHD-PCI and CHD-CABG groups, respectively, and 45 and 16 severe CRS events occurred in the CHD-PCI and CHD-CABG groups, respectively. Our multivariable analysis demonstrated that the incidence of CRS in the CHD-CABG group was significantly higher than that in the CHD-PCI group (aHR: 1.196, 95% CI: 1.064-1.280, *P* = 0.0402), but the two groups had similar incidence rates of severe CRS (aHR: 0.795, 95% CI: 0.456-1.388, *P* = 0.5534). Subgroup analyses revealed that the association between CHD severity and CRS development was more significant among men (*P* = 0.0016). In conclusion, we determined that severe CHD treated with CABG was associated with a higher incidence of subsequent CRS, and this association was more prominent among men.

## Introduction

Coronary heart disease (CHD) is characterized by coronary artery stenosis and subsequent coronary blood flow reduction and myocardial ischemia 1. The incidence of CHD is lower among women; nevertheless, the prognosis for women with CHD is poorer than that for men with CHD 2, 3. CHD can be managed through medical treatment, including the administration of antiplatelet and antihypertensive drugs and anticoagulants 1. Treating advanced CHD involving main coronary artery obstruction necessitates the use of percutaneous coronary intervention (PCI) with a coronary artery bypass graft (CABG) to restore the impaired coronary circulation 4-6.

In addition to affecting the myocardium, CHD contributes to the occurrence of comorbidities outside the heart 7, 8. For example, CHD was reported to be significantly associated with hypertension 9 and was determined to be correlated with metabolic syndrome 10. Moreover, patients with periodontitis were indicated to be at a significant risk of CHD episodes 11. High CHD severity levels were also reported to be associated with a reduction in subfoveal choroidal thickness and an impairment of the choroidal vasculature 12.

Chronic rhinosinusitis (CRS) is a chronic inflammatory disorder of the nasal and paranasal mucosa that often involves the formation of nasal polyps 13. CRS had been demonstrated to be associated with several inflammatory diseases, including asthma and dry eye disease 13, 14. Moreover, Wang et al., reported that CRS patients were at higher risk for acute myocardial infarction occurrence 15. Wu and co-workers highlighted that CHD is considered a comorbid medical disorder for sinusitis patients 16. However, the association between CHD and CRS has yet to be investigated. Because patients with CHD and those with CRS present with inflammatory responses, an association may exist between both conditions.

To fill the aforementioned research gap, the present study investigated the possible association between CHD severity and subsequent CRS by using data from Taiwan's National Health Insurance Research Database (NHIRD). The study determined CHD severity and CRS severity levels on the basis of associated surgical procedures.

## Materials and Methods

### Data Source

This study adhered to the guidelines of the Declaration of Helsinki. The study was approved by the National Health Insurance Administration of Taiwan and the Institutional Review Board of Chung Shan Medical University Hospital (CS1-23044). The requirement for informed consent was waived by these administrative bodies. Taiwan's NHIRD contains claims data from Taiwan's National Health Insurance system. This database contains the medical records of 23 million Taiwanese patients. For this study, patient data for the period from January 1, 2014, to December 31, 2020, were included for analysis. The available patient data included *International Classification of Diseases, Ninth Revision* (*ICD-9*) and *International Classification of Diseases, Tenth Revision* (*ICD-10*) diagnostic codes, age, sex, place of residence, education level, laboratory exam codes, medical department visit records, imaging exam codes, surgical codes, procedure codes, and Anatomical Therapeutic Chemical (ATC) codes for medical prescriptions.

### Patient Selection

**Figure 1:**
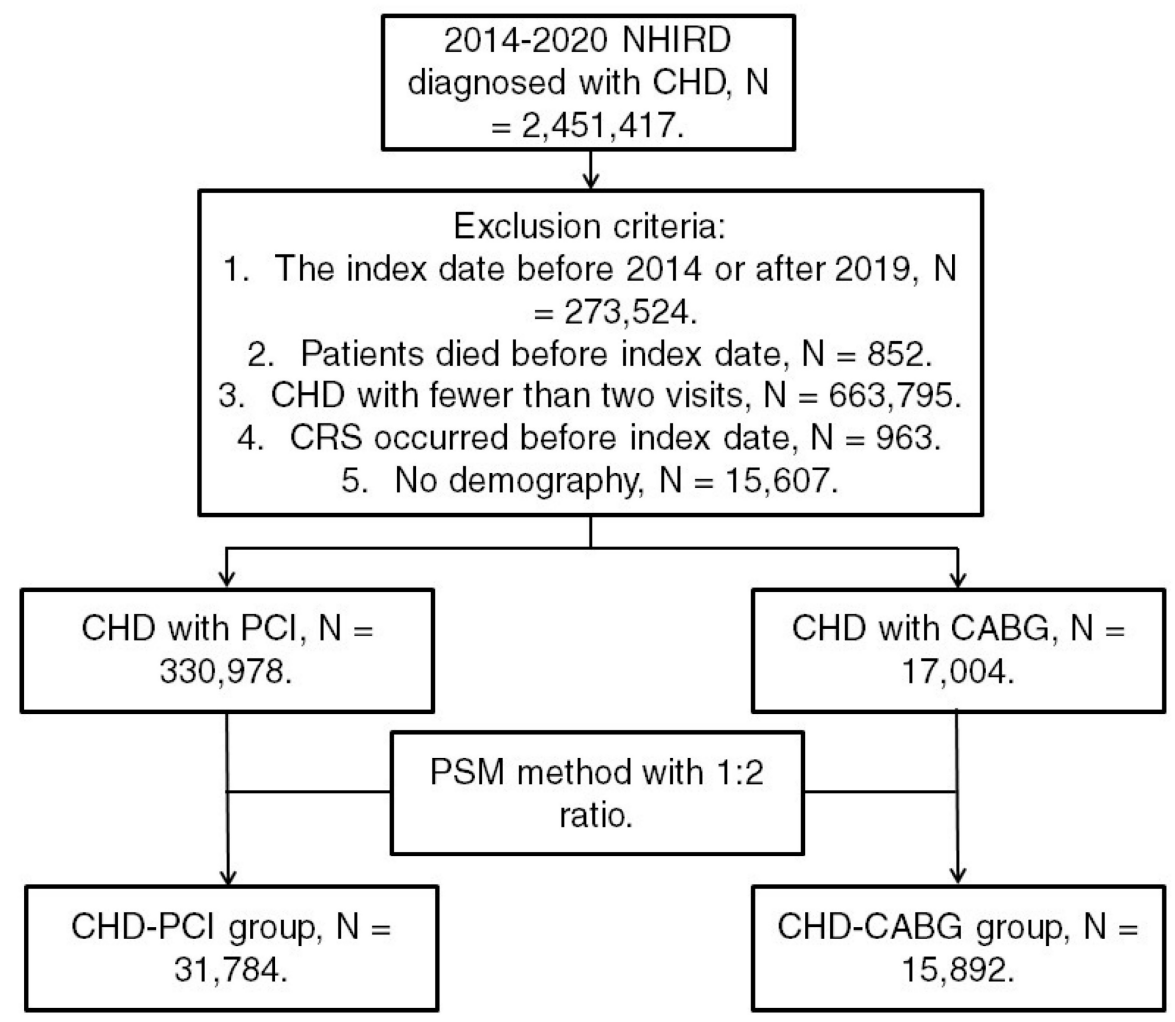
Flowchart of patient selection. NHIRD: National Health Insurance Research Database; CHD: coronary heart disease; N: number; CRS: chronic rhinosinusitis; PCI: percutaneous coronary intervention; CABG: coronary artery bypass graft; PSM: propensity score matching

Patients from the NHIRD were considered to have CHD and included in the study if they met the following criteria: (1) receiving a CHD diagnosis based on *ICD-9* or *ICD-10* codes during 2014-2019; (2) undergoing complete blood cell count, cholesterol, triglyceride, high-density lipoprotein, low-density lipoprotein, white blood cell differentiation, cardiac angiography, and electrocardiography tests before CHD diagnosis; and (3) undergoing follow-up assessments in the internal medicine, family medicine, or cardiovascular department for >2 months. The index date was set as 6 months after CHD diagnosis. Patients were excluded if they met the following criteria: (1) having an index date before 2015 or after 2019, (2) having died before the index date, (3) undergoing fewer than two follow-up assessments for CHD in a medical department, (4) having had CRS before the index date, and (5) having no available demographic data. We categorized CHD severity on the basis of the type of surgery used for CHD treatment; specifically, CHD was categorized as severe if treated using CABG surgery and as mild if treated using PCI. Moreover, we matched each patient who received CABG surgery for CHD with two patients who received PCI surgery for CHD by using propensity score matching (PSM) adjusted for demographics, systemic diseases, and medical prescriptions. Accordingly, after the PSM process, we obtained a total of 31,784 patients who received PCI surgery (the CHD-PCI group) and 15,892 patients who received CABG surgery (the CHD-CABG group). Figure 1 illustrates the patient selection flowchart.

### Primary Outcome

The primary outcome was the presence of CRS that met the following criteria: (1) CRS diagnosed using relevant *ICD-9* or *ICD-10* diagnostic codes, (2) CRS diagnosed after computed tomography and endoscopic examination in accordance with examination codes, and (3) CRS diagnosed by an otorhinolaryngologist. Severe CRS was defined as CRS that met the aforementioned criteria and required functional endoscopic sinus surgery. Only CRS episodes that occurred after the index date were considered in the determination of the primary outcome.

### Demographic and Systemic Confounding Factors

To reduce the effect of confounding factors on the CRS development, we adjusted for the following demographic characteristics, systemic disorders, and prescriptions in our multivariable analyses: age, sex, occupation, hypertension, hyperlipidemia, diabetes mellitus, peripheral vascular disease, cerebrovascular disease, rheumatoid arthritis, systemic lupus erythematosus, Sjögren syndrome, systemic corticosteroids, clopidogrel, aspirin, alpha blockers, beta blockers, calcium channel blockers, angiotensin receptor blockers, angiotensin converting enzyme inhibitors, and statins. The presence of these confounding factors was assessed using *ICD-9*/*ICD-10* codes, insurance codes, and ATC codes in the patients' records. To ensure that these confounding factors had the potential to sufficiently influence CRS development, only comorbidities and prescriptions that persisted for >2 years before the index date were considered confounding comorbidities or prescriptions. The patients in our cohort study were observed until the appearance of CRS, withdrawal from the Taiwan National Health Insurance program, or December 31, 2020 (the end of the follow-up period in this study).

### Statistical Analysis

All statistical analyses were conducted using SAS (version 9.4; SAS Institute, Cary, NC, USA). Descriptive analyses were used to compare demographic characteristics, comorbidities, and medical prescriptions between the CHD-PCI and CHD-CABG groups, and the absolute standardized difference (ASD) was used to compare the distribution of confounding factors between the CHD-PCI and CHD-CABG groups; an ASD value of >0.1 was considered to indicate a significant difference. Furthermore, Cox proportional hazards regression was used to calculate and compare adjusted hazard ratios (aHRs) and 95% confidence intervals (CIs) for CRS and severe CRS between the CHD-PCI and CHD-CABG groups. The effects of age, sex, occupation, systemic comorbidities, and medical prescriptions were adjusted for in the Cox proportional hazards regression. We conducted subgroup analyses by dividing the patients with CHD into subgroups according to age and sex; we then used Cox proportional hazards regression to determine and compare the incidence of CRS and severe CRS in the CHD subgroups. In addition, interaction tests were used to analyze differences in the incidence of CRS and severe CRS between the subgroups. A *P* value of <0.05 indicated significance.

## Results

Table 1 presents the characteristics of the CHD-PCI and CHD-CABG groups. The two groups had similar sex (ASD: 0.0000) and age (ASD: 0.0017) distributions, which can be attributed to the use of PSM. Moreover, the two groups did not differ significantly in terms of occupation, systemic comorbidities, or medical prescriptions (all ASD < 0.1; Table 1).

Throughout the follow-up period (2014-2020), 813 and 482 episodes of CRS occurred in the CHD-PCI and CHD-CABG groups, respectively. Our multivariable analysis demonstrated that the incidence of CRS in the CHD-CABG group was significantly higher than that in the CHD-PCI group (aHR: 1.196, 95% CI: 1.064-1.280, *P* = 0.0402). Furthermore, a total of 45 and 16 severe CRS events occurred in the CHD-PCI and CHD-CABG groups, respectively. The risks of severe CRS were similar between the CHD-PCI and CHD-CABG groups (aHR: 0.795, 95% CI: 0.456-1.388, *P* = 0.5534; Table 2).

Our age-based subgroup analysis revealed that among patients aged <60 years, the risk of CRS was significantly higher in the CHD-CABG population than in the CHD-PCI population (aHR: 1.180, 95% CI: 1.119-1.244). However, the age-based subgroups had similar risks of CRS (*P* = 0.8182). Furthermore, our sex-based subgroup analysis indicated that among male patients, the CHD-CABG population exhibited a higher incidence of CRS than did the CHD-PCI population (aHR: 1.206, 95% CI: 1.007-1.243), and the correlation between CHD severity and CRS development was significantly higher among male patients (*P* = 0.0016). The occurrence of severe CRS did not differ between the subgroups (both *P* < 0.05; Table 3).

## Discussion

This study determined that the CHD-CABG group had a higher risk of CRS than did the CHD-PCI group. Moreover, the CHD-PCI and CHD-CABG groups had similar incidence rates of severe CHD treated using functional endoscopic sinus surgery. The study also determined that the association between severe CHD and subsequent CRS was more prominent in male patients.

Several pathways contribute to the development and progression of CHD 17. Inflammation is a major mechanism of CHD development, and CHD was reported to be associated with relatively high inflammatory cytokine levels 18. A previous study revealed that patients with CHD had elevated lipoprotein-associated phospholipase A2 and C-reactive protein levels 19. Additionally, the neutrophil-to-lymphocyte ratio, the platelet-to-lymphocyte ratio, and C-reactive protein levels can predict CHD severity 20. Atherosclerotic plaques also constitute a major factor contributing to CHD development; such plaques are caused by macrophages and high expression of vascular cell adhesion molecules and matrix E-selectin 17. In addition to inflammation, elevated serum lipid levels can contribute to the risk of CHD; this risk can be attenuated by using statins to lower serum low-density lipoprotein concentrations 21. CRS is also associated with inflammation; specifically, CRS is associated with relatively high expression levels of genes producing interleukin and intercellular adhesion molecules 22. Moreover, CRS involves local aggregation of immune cells such as eosinophils, natural killer cells, and neutrophils in nasal polyps 23. Apart from molecular mechanisms contributing to CHD development, conditions such as obesity and asthma are commonly associated with CHD or CRS 24-27. The advancement of CHD may be associated with the severity of subsequent CRS, and this is attributable to their similar pathophysiology and associated comorbidities 17, 24-28. Our findings support this possible association.

Our study revealed that severe CHD was associated with a higher incidence of subsequent CRS. Previous studies have reported cases of nasal diseases in patients with CHD 29, 30. However, no large-scale study with an adequate sample size has investigated the association between CHD and nasal diseases. To the best of our knowledge, our study is the first to demonstrate a positive association between CHD severity and CRS development. To exclude the influence of preexisting CRS on our findings, we excluded patients with CRS that occurred before CHD diagnosis or within 6 months after CHD diagnosis; moreover, we adjusted for multiple risk factors for CRS, including age, sex, and systemic inflammatory disorders, in our Cox proportional hazards regression 2, 24, 31. Despite these adjustments, we observed that the association between CHD severity and CRS occurrence still remained significant. Consequently, CHD severity may be an independent risk factor for subsequent CRS. A previous study indicated that the severity of CHD was associated with the presence of inflammatory respiratory diseases such as chronic obstructive pulmonary disease 32. Accordingly, we may reasonably assume that CHD severity can affect the occurrence of other inflammatory diseases in the respiratory tract, such as CRS. We observed that the incidence of severe CRS requiring functional endoscopic sinus surgery did not differ significantly between the CHD-PCI and CHD-CABG groups. This phenomenon can be attributed to two possible reasons. First, the increase in inflammation occurring in severe CHD may be inadequate to trigger a significant progression of CRS because other factors are key to the development of severe CRS. Second, the numbers of patients with severe CRS were low in both groups, which may have led to statistical bias.

In our subgroup analyses, the age-based subgroups of patients with CHD had similar risks of CRS. However, our sex-based subgroup analysis revealed that the correlation between CHD severity and CRS occurrence was significantly higher among male patients than among female patients. A previous study reported age to be a risk factor for CHD 24; however, another study indicated that age was not a risk factor for CRS, showing that the severity of CRS was greater among young individuals 33. The correlation between severe CHD and subsequent CRS development in the present study was significant but did not differ by age. However, previous studies have reported that the male sex was associated with a relatively high prevalence of CHD and CRS, rendering the male sex a prominent risk factor for both CHD and CRS 2, 34. The influence of severe CHD may be greater in patients at a high risk of CRS, including men 2, 34, and this assumption is supported by our findings of a stronger correlation between severe CHD and CRS among male patients. We observed nonsignificant associations between severe CRS and CHD severity in all age- and sex-based subgroups, and these observations were noted to be consistent with our overall results.

The prevalence of CHD is >6% globally and is as high as 30% in Northern European men 24, 35. CHD is the second leading cause of all-cause mortality in the United Kingdom 36 and the leading cause of death in the United States, despite a reduction in the mortality rate associated with CHD 37. Additionally, CRS is globally prevalent and is the most common chronic disease in the United States 13. Although death directly caused by CRS is rare, CRS adversely affects daily life, and many patients cannot fully recover from CRS, leading to a substantial economic burden 25. Both CHD and CRS affect many individuals and can lead to substantial health problems 13, 38; hence, identifying any correlation between them is crucial.

This study has some limitations. First, we used data from the NHIRD, which does not contain real medical records. Therefore, we could not obtain or analyze data on several crucial factors such as imaging results for CHD, serum lipid levels for CHD, degrees of arterial stenosis in CHD, treatment details for CHD, the prognosis of CHD, nasal imaging results for CRS, surgical outcomes for CRS, and therapeutic outcomes for CRS. Second, we applied a retrospective study design, and the health of the patients may have changed; nevertheless, PSM was used to minimize the influence of this. Third, we determined disease severity by considering the treatment procedure used rather than conducting a clinical evaluation, which may have engendered a substantial bias because PCI can be used to treat advanced coronary artery stenosis with acceptable outcomes 39, 40. Finally, we excluded a considerable number of patients with CHD treated with PCI during the matching process. Nonetheless, our sample size is not smaller than those in previous population-based studies 14, 24; hence, the influence of this limitation might be minimal.

In conclusion, we observed that severe CHD treated with CABG was associated with a higher incidence of subsequent CRS when compared with mild CHD treated with PCI. Accordingly, CRS-related examinations may be recommended for individuals with severe CHD undergoing surgical management. Further large-scale prospective research is warranted to determine whether the severity of CHD influences therapeutic outcomes for CRS of varying severity.

## Figures and Tables

**Table 1:** Characteristics of patients receiving percutaneous coronary intervention and coronary artery bypass graft surgery

Character	CHD-PCI group (N = 31,784)	CHD-CABG group (N = 15,892)	ASD
Sex			0.0000
Male	22287 (70.12%)	11143 (70.12%)	
Female	9497 (29.88%)	4749 (29.88%)	
Age			0.0017
<40	801 (2.52%)	267 (1.68%)	
40-49	2689 (8.46%)	1049 (6.60%)	
50-59	6061 (19.07%)	3145 (19.79%)	
60-69	9538 (30.01%)	5839 (36.74%)	
≥70	12695 (39.94%)	5592 (35.19%)	
Occupation			0.0025
Government employee	1491 (4.69%)	623 (3.92%)	
Worker	16375 (51.52%)	8181 (51.48%)	
Farmer and fisherman	7857 (22.47%)	2520 (15.86%)	
Low-income	346 (1.09%)	323 (2.03%)	
Others	5715 (20.23%)	4245 (26.71%)	
Co-morbidities			
Hypertension	24582 (77.34%)	12812 (80.62%)	0.0038
Diabetes mellitus	14567 (45.83%)	9462 (59.54%)	0.0254
Hyperlipidemia	18791 (59.12%)	10056 (63.28%)	0.0107
Cerebrovascular disease	2972 (9.35%)	2012 (12.66%)	0.0331
Peripheral vascular disease	1405 (4.42%)	828 (5.21%)	0.0076
Rheumatoid arthritis	496 (1.56%)	138 (0.87%)	0.0198
Systemic lupus erythematosus	130 (0.41%)	99 (0.62%)	0.0005
Sicca/Sjogren syndrome	407 (1.28%)	133 (0.84%)	0.0010
Medications			
Systemic corticosteroids	7835 (24.65%)	10343 (32.54%)	0.0145
Aspirin	20396 (64.17%)	22621 (71.17%)	0.0397
Clopidogrel	6954 (21.88%)	11204 (35.25%)	0.0627
Alpha-blockers	2762 (8.69%)	3585 (11.28%)	0.0288
Beta-blockers	17570 (55.28%)	19979 (62.86%)	0.0186
CCB	18241 (57.39%)	16315 (51.33%)	0.0130
ACEi	12482 (39.27%)	15485 (48.72%)	0.0228
ARB	2552 (8.03%)	3433 (10.80%)	0.0084
Statin	16518 (51.97%)	21454 (67.50%)	0.0386

N: number; CHD: coronary heart disease; PCI: percutaneous coronary intervention; CABG: coronary artery bypass graft; ASD: absolute standardized difference; CCB: calcium channel blocker; ACEi: angiotensin converting enzyme inhibitor; ARB: angiotensin receptor blocker

**Table 2:** Risk of chronic rhinosinusitis in patients with coronary heart disease treated with percutaneous coronary intervention or coronary artery bypass grafts

Event	CHD-PCI group	CHD-CABG group	P value
**CRS**			
Person-months	1098512	422078	
Event	813	482	
Crude HR (95% CI)	Reference	1.512 (1.246-1.835)*	
aHR (95% CI)	Reference	1.196 (1.064-1.280)*	0.0402*
**Severe CRS**			
Person-months	1002136	415645	
Event	45	16	
Crude HR (95% CI)	Reference	0.801 (0.463-1.385)	
aHR (95% CI)	Reference	0.795 (0.456-1.388)	0.5534

CHD: coronary heart disease; PCI: percutaneous coronary intervention; CABG: coronary artery bypass graft; CRS: chronic rhinosinusitis; HR: hazard ratio; CI: confidence interval; aHR: adjusted hazard ratio. The results obtained from the Cox regression model.* significant difference between groups

**Table 3:** Subgroup analyses of chronic rhinosinusitis incidence by age and sex

Subgroup	aHR	95% CI	Interaction P value
**CRS**			
Age			0.8182
<60	1.180	1.119-1.244	
≥60	1.125	0.899-1.409	
Sex			0.0016*
Male	1.206	1.007-1.243	
Female	0.755	0.483-1.382	
**Severe CRS**			
Age			0.2575
<60	1.097	0.933-1.291	
≥60	0.571	0.140-2.332	
Sex			0.3346
Male	0.803	0.408-1.454	
Female	0.555	0.383-2.182	

CRS: chronic rhinosinusitis; aHR: adjusted hazard ratio; CI: confidence interval. The results obtained from the Cox regression model.* significant difference between groups.
